# Transfusion of red cells in hematopoietic stem cell transplantation (*TRIST*): study protocol for a randomized controlled trial

**DOI:** 10.1186/1745-6215-12-207

**Published:** 2011-09-21

**Authors:** Jason Tay, Alan Tinmouth, Dean Fergusson, David Allan

**Affiliations:** 1 The Ottawa Hospital Blood and Marrow Programme, The Ottawa Hospital, Ottowa, Canada; 2 Transfusion Research Centre, University of Ottawa and Clinical Epidemiology Program, Ottawa Hospital Research Institute, The Ottawa Hospital, Ottowa, Canada

**Keywords:** Hematopoietic Stem Cell Transplant, Red cell transfusion, Erythrocyte, Triggers, Randomized Clinical Trial, Pilot

## Abstract

**Background:**

Insight regarding transfusion practices in Hematopoietic Stem cell Transplantation (HSCT) are lacking and the impact of red cell transfusion in this high risk group on outcomes following HSCT are not well appreciated. Red blood cell transfusion can be life-saving, however, liberal use of transfusion in critically ill patients failed to demonstrate significant clinical benefit. A large number of other observational studies have also demonstrated an association between red blood cell transfusions and increased morbidity such as infections and multi organ failure as well as increased mortality. The role of red cell transfusion on the clinical outcomes observed in patients undergoing HSCT remains poorly understood and a prospective randomized study of transfusion is required to gain insight and knowledge on best transfusion practices in this high risk population.

**Methods:**

This report describes the design and methodological issues of a randomized pilot study evaluating red cell transfusion triggers in the setting of Hematopoietic Stem Cell Transplantation. This study has been funded by a peer review grant from the Canadian Blood Services and is registered on Clinicaltrials.gov NCT01237639.

**Results:**

In 3 Canadian centres, 100 patients undergoing Hematopoietic Stem Cell Transplantation will be randomized to either a restrictive (target hemoglobin of 70-90 g/L) or liberal (target hemoglobin of 90-110 g/L) red cell transfusion strategy, based daily hemoglobin values up to 100 days post-transplant. The study will stratify participants by centre and type of transplant. The primary goal is to demonstrate study feasibility and we will collect clinical outcomes on 1) Transfusion Requirements, 2) Transplant Related Mortality, 3) Maximum grade of acute Graft versus Host Disease, 4) Veno-occlusive Disease, 5) Serious Infections, 6) Bearman Toxicity Score, 7) Bleeding, 8) Quality of Life, 9) Number of Hospitalizations and 10) Number of Intensive Care Unit (ICU) Admissions.

**Conclusion:**

Upon completion, this pilot trial will provide preliminary insight into red cell transfusion practice and its influence in hematopoietic stem cell transplant outcomes. The results of this trial will inform the conduct of a larger study.

## 1. Background

Evidence based transfusion practices in Hematopoietic Stem cell Transplantation (HSCT) are lacking and the impact of red cell transfusion on outcomes following HSCT are not well appreciated. Although, red blood cell transfusion can be life-saving, liberal use of transfusion in critically ill patients failed to demonstrate significant clinical benefit in a landmark Canadian study [1]. In fact, increased 30 day mortality was observed in a subset of patients transfused at a higher hemoglobin level and red blood cell transfusion may contribute to impaired wound healing in various other settings. In one single-centered retrospective study, patients undergoing allogeneic transplantation who had reduced hemoglobin at the time of transplant received a greater number of red cell transfusions and had increased transplant-related mortality [2]. Similar results were obtained in a multi-centered study coordinated by one of the authors [3] and reduced hemoglobin prior to autologous transplantation is associated with greater number of transfused red cell units and increased toxicity [4]. Moreover, a recent study suggests that red cell transfusion in cancer patients is associated with increased risk of thrombosis and in-hospital mortality [5]. Our systematic search of the literature on transfusion strategies in HSCT reveals a paucity of knowledge, in particular an absence of definitive clinical studies.

The most compelling evidence that red cell transfusion may be harmful is derived from the TRICC study [1]. In 838 critically ill patients randomized to a restrictive transfusion strategy (target hemoglobin of 70-90 g/L with a hemoglobin transfusion threshold of 70 g/L), or a liberal transfusion strategy (target hemoglobin of 100-120 g/L with a hemoglobin transfusion threshold of 100 g/L), patients in the restrictive arm had lower hemoglobin levels (received fewer red cell transfusions, and had lower mortality at 30 days). The results from this seminal trial demonstrated that a restrictive red blood cell transfusion strategy reduces red cell transfusion requirements and is at least as safe as, and possibly superior to, a more liberal approach for critically ill adults. These data showing that restrictive transfusion triggers can reduce red cell transfusion use without significantly increasing adverse clinical outcomes have been replicated in pediatric [6] and neonatal ICU [7] patients. Additionally, in a recent systematic review of 45 observational trials, red cell transfusions were associated with an increased risk of infection, multi-organ failure, and death [8].

With regards to the HSCT population, Xenocostas et al [2] reported that patients undergoing allogeneic transplantation required an average of 6.8 ± 6.4 units of red cells between 0 - 60 days after transplant and the frequency of red cell transfusion decreased significantly thereafter. Increased numbers of red cell transfusions were associated with the before-transplant hemoglobin level, major ABO mismatch between donor and recipient, transplantation in patients with more advanced disease, with the use of unrelated compared with related donors, older patient age and female gender. The before transplant hemoglobin level was also associated with increased mortality within 6 months of transplant in multivariate regression analysis. The association of reduced hemoglobin prior to allogeneic HSCT with increased red cell transfusion requirements and increased 180-day mortality was confirmed in a multi-centered analysis [3]. With regards to patients undergoing autologous transplantation, reduced hemoglobin prior to HSCT was associated with increased red cell transfusion requirements in a study recently published by our group. Furthermore, reduced hemoglobin on the day of stem cell collection was associated with lower levels of vascular progenitor cells in the stem cell collection and increased organ-specific toxicity following high-dose chemotherapy [4].

Conceptually, higher hemoglobin levels would correlate with better performance status and/or quality of life. Indeed, there have been positive associations noted between a higher hemoglobin and quality of life in a variety of clinical populations [9-13]. Nonetheless, there is a paucity of data in the transplant literature reviewing the impact of hemoglobin levels and quality of life. However, a reduced hemoglobin level prior to transplantation has been associated with increased post transplant mortality and toxicity [2]. It remains unclear whether improvements in hemoglobin levels prior and/or during transplant would abrogate these negative outcomes. The role of red cell transfusion on the clinical outcomes observed in patients undergoing HSCT remains poorly understood and a prospective randomized study of transfusion is required to gain insight and knowledge on best transfusion practices in this high risk population.

In preparation for our trial, all adult transplant centres associated with the Canadian Blood and Marrow Transplant Group (n = 15) were asked to respond to a survey regarding transfusion thresholds for red cells. Fourteen centres routinely transfuse 2 units of Packed Red Blood Cells (PRBC) when the hemoglobin < 80 g/L and/or in response to patient specific indications. One centre transfuses PRBCs when the hemoglobin < 70 g/L and/or response to specific circumstances. Importantly, the rationale for the near universal adoption of red cell transfusion trigger of 80 g/L (personal communication with Canadian transplant Directors) remains unclear. There appears to be a reluctance to fully embrace a hemoglobin trigger of 70 g/L as suggested by the TRICC study, recognizing that there are differences between ICU and HSCT patients. This may have lead to a "middle of the road" approach, with centres selecting a trigger of 80 g/L with historical red cell transfusion triggers as high as 100 g/L.

Our coordinating centre has historically adopted a red cell transfusion trigger of 80 g/L and we recently conducted an audit of our transfusion practices for the year 2009. Despite our established trigger of 80 g/L, red cell transfusions above this trigger occurred in 50% of all transplant patients ever requiring red cells within the 1^st ^100 days of the transplant. Further, 20% of all red cell transfusion episodes occurred above the stated trigger. This phenomenon has also been observed in other Canadian centres (personal communication).

No centre has clearly embraced a liberal transfusion strategy and only 1 centre has embarked on a more restrictive strategy. This indicates some relative uncertainty with regards to the benefits and harms of PRBC transfusion in the HSCT population. If we assume that the current threshold may be associated with some potential for harm and some potential for benefit, it is reasonable to consider whether transfusion at 70 g/L is better or worse than transfusing at 90 g/L.

This report describes our design and methods of TRIST, an open labeled multi-center pilot randomized controlled trial in adult patients undergoing HSCT to assess if restrictive strategy to maintain a hemoglobin greater than 70 g/L compared to a more liberal strategy of maintaining hemoglobin greater than 90 g/L is safe and associated with reduced transplant-related mortality following HSCT. This trial will focus on feasibility outcomes in order to facilitate the planning and conduct of a definitive phase III trial.

## 2. Methods

### 2.1 Trial Overview

TRIST is an open-labeled multi-center pilot parallel-arm randomized controlled trial, where participants will be stratified by centre (3 centres) and type of HSCT (either autologous or allogeneic). We will recruit patients over a 2 year period and follow each participant for a minimum of 100 days. Following a screening visit to confirm eligibility, patients will be randomized with subsequent study assessments to occur in concert with routine transplant care on Days +7, +14, +28, +60, +100 (where Day 0 is defined as the 1 ^st ^day of stem cell infusion). TRIST has been funded by a peer reviewed grant by the Canadian Blood Services (R & D Intramural Grants Competition 2010) and registered on the National Institute of Health, Clinicaltrials.gov registry (NCT01237639).

### 2.2 Study participants

Study participants will be recruited from 3 Canadian adult HSCT centers. They will be adult recipients of either an autologous or allogeneic HSCT. We chose to study both populations to 1) better appreciate the potential benefits and/or harms with our intervention in these 2 different types of HSCT and 2) to help define a feasible primary endpoint for a definitive clinical trial. Specific inclusion and exclusion criteria are listed in Table 1.

**Table 1:** Inclusion and Exclusion Criteria

	*Inclusion Criteria*
1	Males or females aged 18 years or older who are undergoing either an autologous or allogeneic HSCT.

2	The indications for HSCT may include, but not limited to the following diseases: a. Acute Leukemia, myeloid, lymphoid or biphenotypic in 1^st^, 2^nd ^remission or in relapse b. Chronic Myeloid Leukemia in chronic, accelerated or blast phase c. Chronic Lymphocytic Leukemia d. Myelodysplastic Syndrome e. Myeloproliferative Disorder f. Lymphoma g. Myeloma

3	All study patients must provide consent at least 1 day prior to scheduled HSCT and provide written informed consent.

	***Exclusion Criteria***

1	Pregnant or lactating at the time of enrollment.

2	Already received red cell transfusion after conditioning chemotherapy for HSCT but prior to enrollment.

3	Unable/unwilling to providing informed consent.

4	Geographically inaccessible

### 2.3 Allocated treatment strategies

Eligible participants will have daily (morning) laboratory investigations, including a Complete Blood Count (CBC) performed as standard of care. The recipient will be randomized to one of two red cell transfusion strategies, to be carried out based on daily morning CBCs (The transfusion strategy will be carried out from Day 0 until Day 100 post stem cell infusion/transplant):

1. Liberal Strategy (Red cell transfusion trigger of 90 g/L): Participants will receive 2 units of packed red blood cells if the Hemoglobin (Hb) level is < 90 g/L, based the day's CBC to target a Hb level of 90-110 g/L

2. Restrictive Red cell Transfusion Strategy (Red cell transfusion trigger of 70 g/L): Participants will receive 2 units of packed red blood cells if the Hb level is < 70 g/L, based on the day's CBC to target a Hb level of 70-90 g/L

Transfusion(s) of red cells outside the red cell transfusion strategy is permitted when clinically indicated by the treating physician.

### 2.4 Randomization and treatment allocation

The randomization process will consist of a computer generated random list of permutated blocks of 2 and 4, stratified by type of HSCT (autologous or allogeneic) and by centre (3 centres). The randomization sequence will be determined by the Ottawa Hospital Research Institute-Methods Centre statistician, independent from the study. After screening the patient for eligibility and obtaining informed consent, the study nurse will randomize the patient using the number in the next chronological order from the randomization lists provided ahead of time by the Methods Centre. This randomization list is secured within a password protected SQL server. The participant's assignment is only known once all the inclusion criteria are reviewed and "checked-off" online. The local research coordinator/nurse will then identify the transfusion intervention assignment of the participant to the HSCT team.

### 2.5 Medical Management

In addition to the primary intervention of assignment of either a restrictive or liberal transfusion strategy, all other HSCT support care will be according to local institutional practice. This will include, but not limited to the use of antibiotics, analgesia, intravenous fluids, growth factors and blood products (not including red cells).

### 2.6 Withdrawal from study

Participants may withdraw from the study at any time and for any reason, or maybe withdrawn at the treating physician's discretion in the event of intolerance to transfusion assignment, adverse events, or administrative reasons.

### 2.7 Study Outcome measures: Feasibility

The primary goal of this pilot study is to demonstrate feasibility. We will (1) identify logistical issues related to protocol implementation. Further, we will evaluate feasibility of (2) recruitment rates, (3) randomization implementation strategy, (4) data collection of clinical outcomes and (5) define the sample size required for a definitive trial. To evaluate the feasibility of recruitment, we will seek to demonstrate the ability to recruit 3 patients per centre per month (based on investigator consensus).

We also seek to document compliance with the assigned red cell transfusion threshold. Compliance will be assessed by the number of protocol violations for red cell transfusions (participants receiving PRBCs above or below the assigned transfusion trigger in the absence of a clinical rationale e.g. angina). We have arbitrarily defined compliance (based on total PRBC transfusion episodes) to the assigned red cell transfusion threshold as follows: Excellent (≤5% violations), Acceptable (between 5% and 20% violations), Poor (≥20% violations). Further, the mean difference of pre-transfusion Hemoglobin will be calculated, in order to assess adherence to the protocol.

### 2.8 Study Outcome measures: Clinical Outcomes

The following clinical outcomes (see Table 2 for definitions) will be evaluated: 1) Transfusion Requirements (red cells, platelets and plasma), 2) Transplant Related Mortality, 3) Maximum grade of acute Graft versus Host Disease [14] 4) Veno-occlusive Disease [15], 5) Serious Infections [16], 6) Bearman Toxicity Score [17], 7) Bleeding [18], 8) Quality of Life [19,20], 9) Number of Hospitalizations and 10) Number of ICU Admissions. Further, we will document any transfusion reactions and the duration of storage of each red cell unit prior to infusion.

**Table 2:** Definitions and Description of Clinical Outcomes

*Transfusion Requirements*	This defined as the number of transfusions received by the recipient between the 1^st ^day of Stem cell reinfusion (Day 0) and Day 100 post Hematopoietic Stem Cell Transplantation.
***Transplant Related Mortality***	This is defined as the incidence of death from any cause at 100 days post Hematopoietic Stem Cell Transplantation.

***Maximum grade of acute Graft versus Host Disease (GVHD)***	The incidence of acute GVHD up to Day 100 will be compared. Acute GVHD will be graded according to the Przepiorka Criteria [14].

***Serious Infection***	Clinically important infections will be ascertained using Centre for Disease Control criteria [16]. Infections will include: serious infections such as nosocomial pneumonia; deep tissue infections (peritonitis, mediastinitis) and bacteremia from organisms not considered normal skin flora, and judged important enough to treat by the attending team. All grade 4 and 5 infections (according to the Common Terminology for Adverse Events v.4) will be recorded in the Data Collection Forms. In addition, the following information will also be recorded in the Data Collection Forms: Type of organism (bacterial, viral, fungal or protozoal) (suspected or documented).

***Bearman Toxicity Score***	This is a validated scoring system [17] to assess toxicity during HSCT. In this system, grade I toxicity is reversible without treatment and grade 2 is not life threatening, but requires treatment. Grade 3 requires life-support intervention and grade 4 is fatal. Regimen-related toxicity in each organ system was scored as the highest grade achieved in that organ system through day 28, except that deaths occurring after day 28 as a result of regimen-related toxicity occurring before day 28 are also scored as grade 4. Adverse events that could be attributed to infection (culture-documented), bleeding or other medications are not scored as regimen-related toxicity. The maximum toxicity is the highest grade recorded in any individual organ system and the cumulative toxicity score is the sum of the highest grades recorded for all eightorgan systems.

***Veno-Occlusive Disease***	Diagnosis requires 2 of 3 criteria, occurring within 20 days of transplantation: 1) Bilirubin > 34.2 μmol/L (2 mg/dL), 2) Hepatomegaly or RUQ pain of liver origin and 3) > 2% weight gain due to fluid accumulation [15].

***Bleeding***	The WHO Scale [18] is a credible, standardized and validated scale to assess bleeding. Grade 3 and 4 bleeding will be recorded in the data collection forms.

***Quality of Life***	Both the FACT-BMT [20] and EQ-5D [19] scales will be used to document quality of life. The FACT consists of 5 subscales that measure physical well-being, functional well-being, social/family well-being and emotional well-being. The BMT subscale of the FACT includes additional items specifically designed to test quality of life and symptoms specific to BMT patients. EQ-5D is a standardized measure of health status developed by the EuroQol Group in order to provide a simple, generic measure of health for clinical and economic appraisal.

HSCT: Hematopoietic Stem Cell Transplantation

### 2.9 Blinding

This is an open labeled study. It will be impossible to blind the assigned red cell transfusion trigger to participants and bedside caregivers since the standard of care requires daily knowledge of hemoglobin concentration as part of the participants' overall HSCT care and safety. There will be no specific measures to blind research coordinators and study statisticians, as the clinical outcomes sought as listed in Section 2.8 are "hard" clinical outcomes and assigned primarily by the bedside caregiver. Consequently, the risk of measurement bias is minimal.

### 2.10 Screening, Baseline and Follow-up assessments

Potential participants will be identified by screening at HSCT transplant consultation and/or planning visits. Following written informed consent, demographic information will be collected and participants will be screened for eligibility at the screening visit (see Table 3 for study schedule and procedures at all study visits).

**Table 3:** Schedule and Evaluation of Outcomes

		Study Entry	Scheduled Assessments (± 3 days as inpatient; ± 5 days as outpatient)					
			Day 0	Day 7	Day 14	Day 28	Day 60	Day 100
Demographics/Baseline characteristics	Trial Case Number	x						
	Date of Birth	x						
	Gender	x						
	Indication for HSCT	x						
	Stem cell Source	x						
	CD34 cell count	x						
	Donor Type (allos only)	x						
	HLA compatibility (allos only)	x						
	ABO blood type	x						
	ABO compatibility (allos only)	x						
	CMV compatibility (allos only)	x						
	Karnofsky Functional Scale	x						
	Conditioning regimen	x						
	HCT-CI	x						
	CBC	x	all available values					
Quality of Life Scales	EQ-5D and FACT-BMT†	x		x	x	x	x	x
Transfusion Assessments	Red Cells Transfusion Assessment		As needed					
	Platelets Transfusion Assessment		As needed					
	Plasma and Cryoprecipitate Transfusion Assessment		As needed					
Transplant Assessments	Bleeding Assessment				x	x		x
	Infection Assessment				x	x		x
	VOD Assessment^#^					x		
	Bearman Toxicity Assessment^					x		
	aGVHD*							x
	Admission to ICU		As needed					
	Hospital Admission		As needed					
	Mortality Assessment							x

NB Scheduled Assessments will be required even if the patient is discharged; the assumption is that no transplant toxicities have occurred if the patient is discharged

† Quality of Life Scales may be completed at home to be mailed or handed in

^# ^Presence/Absence within the 1st 21 days of HSCT (by Seattle Criteria) ^ Highest grade to be completed * Highest grade of aGVHD to be completed

HLA: Human Leukocyte Antigen; CMV: Cytomegalovirus; HCT-CI: Hematopoietic Cell Transplantation-Comorbidity Index; CBC: Complete Blood Count;

VOD: Veno-occlusive Disease; aGVHD: acute graft versus host disease; ICU: Intensive Care Unit

### 2.11 Sample Size

This is a pilot study that would inform a definitive trial. The transplant related mortality (TRM) at 100 days (main clinical outcome) is not clearly known for the proposed red cell transfusion triggers/thresholds. We propose a sample size of 50 adults in each treatment group for a total sample size of 100, while stratifying for participants undergoing either an Autologous or Allogeneic HSCT. Preliminary sample size calculations based on the following assumptions, TRM of 15% in the liberal strategy, 10% in the restrictive strategy, α = 0.05 with a total sample size of 100 would yield a power of 7.3%. Nonetheless, the conduct of this pilot trial will allow us to accurately estimate the event rates in all the studied clinical outcomes and suggest feasible clinical outcomes to evaluate.

### 2.12 Data Management

Data Collection is facilitated by paper-based case report forms designed in consultation with Ottawa Hospital Research Institute- Methods Centre. Research Coordinators at each site will complete the case report forms, check them for accuracy and completeness by reviewing source documents and by regular interaction with the transplant team. Finally, the Research Coordinators will fax the case report forms to the coordinating centre (Ottawa) via a dedicated fax machine. Case report forms will be faxed to the Project Management Office at registration and at follow-up time points. Data will be identified by an alphanumeric code only. Data will be entered into a Microsoft Access Database (within a secured SQL server) which exactly reflects the case report forms. Any queries arising from missing data or data anomalies will be resolved.

Source documentation will remain at their respective participating centre's site. The server for the database will be located in the Ottawa Hospital Research Institute under the care of the Methods Centre. Appropriate security measures will be in place such that current Canadian privacy laws are adhered to with respect to security and confidentiality of data, electronic data transmission, data storage and data access. A secure ID and password will be necessary to access the system. Audit trails of entries will be provided. The Project Manager and delegate will be the only individuals that can edit data.

### 2.13 Ethical considerations

This study will be conducted in accordance with the Health Canada's Good Clinical Practice (GCP) guidelines [21], in accordance with the current Declaration of Helsinki and the Tri-Council Policy Statement: Ethical Conduct for Research Involving Humans (TCPS)[22]. The study protocol and amendments and informed consent forms have been approved by the research ethics board of each participating center. Written informed consent will be obtained from each participant.

### 2.14 Study Management

The principal investigator (PI) at each site will oversee the day-to-day management of the trial, resolving questions about eligibility, enrolment, randomization, grading and disposition of toxicities and adverse events, and determination of outcomes questions that need resolution. Further, the trial multi-centre coordinator will provide each centre with initiation visits and at least one monitoring visit to facilitate recruitment and ensure data integrity.

### 2.15 Data Safety and Monitoring Board

The DSMB will be independent and composed of 3 members: an expert in HSCT, an expert in transfusion medicine, and an expert in clinical trials (chair). A DSMB charter will be provided to the DSMB. All serious adverse events will be reported to and reviewed by the DSMB. A DSMB meeting will be conducted once 25 patients have completed their treatment phase of the study. Subsequently, the DSMB will meet after every 25^th ^participant is randomized and reports to the co- PIs. Should safety issues arise that the DSMB feel compromises the trial and/or participant safety, then a meeting will be convened of all co-investigators to consider amending or stopping the trial. DSMB will receive three interim reports and can, at any time, recommend termination of the study in the event that it appears unsafe to continue. There is no stopping rules for this pilot study; the steering committee and DSMB will use their judgment in the event the trial has to be terminated early for safety concerns.

All interim reports (summarized by the study statistician) will include the following aggregated data: 1) Number of patients enrolled, 2) Proportion of patients at 28 days, 3) Proportion of patients at 100 days, 4) Number of autologous and allogeneic HSCTs, 5) Number of Red cell Transfusions, 6) Mean difference of pre-transfusion Hemoglobin (to assess adherence to the protocol), 7) Transfusion Reactions from Red cells, 8) Bleeding as per WHO scale, 9) Veno-occlusive Disease for recipients of Allogeneic HSCT, 10) Assessment of the Bearman Toxicity Score, 11) Acute GVHD for recipients of Allogeneic HSCT, 12) Number of hospitalizations (and length of each admission) from start of conditioning up until 100 days or death, whichever occurs first, 13) Number of ICU Admissions (and length of each admission) from start of conditioning up until 100 days or death, whichever occurs first, 14) Serious Adverse Events, 15) Grade 4 and 5 Infections and 16) Deaths. Further data will be provided to the DSMB when deemed necessary by the DSMB.

The board comprises of Dr. Donald Arnold (Chair), Program Director, Transfusion Medicine McMaster University, Dr. Stephan Couban, HSCT Physician at Dalhousie University and Dr. Lois Shepard, Transfusion Medicine Physician at Queens University.

### 2.16 Statistical Analysis

The number of eligible participants will be documented and the proportion of randomized patients noted monthly. Recruitment at each participating centre will be summarized by the number of randomized participants on a monthly basis. Feasibility of data collection will be assessed by the number of missing or erroneous data at each centre and summarized by proportions.

Clinical outcomes will be collected as described above; they will be compared between the 2 groups (liberal and restrictive red cell transfusion strategy). These will include transfusion requirements (number of PRBC units, platelets and plasma), transplant related mortality at 100 days (yes/no), maximum grade of acute graft versus host disease, incidence of acute graft versus host disease at 100 days, veno-occlusive disease, incidence of grade 4 and 5 infections at 100 days, Bearman toxicity scores, incidence of bleeding, incidence of hospital admissions, incidence of ICU admissions and quality of life.

Comparisons of time to failure endpoints will incorporate Kaplan-Meier probability estimates, log rank testing and, when adjustment of covariates is made, use of the Cox proportional hazards model. Binary/categorical endpoints will be compared between treatment groups using logistic regression. The effect of missing data and outliers will be subjected to sensitivity analysis. The results for each of endpoints will be summarized by a significance test and 95 per cent confidence interval. Analysis of secondary endpoints will be considered exploratory and hypothesis generating. Given that multiple comparisons increase the probability of a Type I error, adjustment of individual statistical tests using a more strict cut point (p < 0.01) will be used to facilitate interpretation. All statistical tests quoted will be 2-tailed. Analysis will follow the intention-to-treat principle. Analyses will be conducted using SAS version 9.2 and/or SPSS.

Sub-group analysis will be conducted as follows: The two treatment groups will be compared among recipients of allogeneic or autologous Hematopoietic Stem cell Transplantation. Results will be considered hypothesis-generating.

## 3. Conclusions

The role of red cell transfusion on the clinical outcomes observed in patients undergoing HSCT remains poorly understood and a prospective randomized study of transfusion is required to gain insight and knowledge on best transfusion practices in this high risk population. While there are clear benefits of red cell transfusion, potential harm has been described in several patient groups. Given the resource utilization and high rate of adverse outcomes in HSCT, our study will provide the framework for better understanding the optimal use of red cell transfusion in this important group of high risk cancer patients. In this study, we aim to determine the feasibility and logistics of conducting a multicenter trial and determine clinical outcome rates in order to plan and execute a prospective definitive randomized study to evaluate the impact of a restrictive and liberal red cell transfusion threshold on transplant outcomes.

## 4. Milestones and current status

This trial was conceived and designed in 2009. Peer reviewed funding through the Canadian Blood Services (Health Canada) intramural grant was sought in 2010, and successfully obtained in 2010. The study began enrolling in March, 2011. Two of 5 invited Canadian Transplant Centres declined participation due to logistical reasons. One center (Saskatoon) is currently in the local ethics approval process and the other (London) has recently enrolled 1 patient. The primary centre (Ottawa) has enrolled 20 patients as of 15 July 2011. Enrollment is reaching its quarter and the DSMB will be reviewing our progress shortly.

## 5. Abbreviations

HSCT: Hematopoietic Stem cell Transplantation; ICU: Intensive Care Unit; PRBC: Packed Red Blood Cells; CBC: Complete Blood Count; TRM: Transplant Related Mortality; DSMB: Data Safety Monitoring Board.

## 6. Conflict of interests

The authors declare that they have no competing interests. The sponsors had no role in study design and preparation of the article, conduct of the study, and in the decision to submit the paper for publication.

## 7. Authors' contributions

All listed authors (JT, AT, DF and DA) were equally involved in the design of the study and original grant submission to the Canadian Blood Services. JT is the principal investigator involved in the protocol development, "write-up" of this manuscript, execution and "day to day" management of this study. Equal input was received from all other listed authors with respect to the above activities. All authors read and approved the final manuscript.
